# Xanthines Studied via Femtosecond Fluorescence Spectroscopy

**DOI:** 10.3390/molecules21121668

**Published:** 2016-12-03

**Authors:** Pascale Changenet-Barret, Lajos Kovács, Dimitra Markovitsi, Thomas Gustavsson

**Affiliations:** 1 LIDYL, CEA, CNRS, Université Paris-Saclay, F-91191 Gif-sur-Yvette, France; pascale.changenet-barret@polytechnique.edu (P.C.-B.); dimitra.markovitsi@cea.fr (D.M.); 2 Department of Medicinal Chemistry, University of Szeged, Dóm tér 8, 6720 Szeged, Hungary

**Keywords:** xanthines, femtosecond spectroscopy, fluorescence, electronic excited states

## Abstract

Xanthines represent a wide class of compounds closely related to the DNA bases adenine and guanine. Ubiquitous in the human body, they are capable of replacing natural bases in double helices and give rise to four-stranded structures. Although the use of their fluorescence for analytical purposes was proposed, their fluorescence properties have not been properly characterized so far. The present paper reports the first fluorescence study of xanthine solutions relying on femtosecond spectroscopy. Initially, we focus on 3-methylxanthine, showing that this compound exhibits non-exponential fluorescence decays with no significant dependence on the emission wavelength. The fluorescence quantum yield (3 × 10^−4^) and average decay time (0.9 ps) are slightly larger than those found for the DNA bases. Subsequently, we compare the dynamical fluorescence properties of seven mono-, di- and tri-methylated derivatives. Both the fluorescence decays and fluorescence anisotropies vary only weakly with the site and the degree of methylation. These findings are in line with theoretical predictions suggesting the involvement of several conical intersections in the relaxation of the lowest singlet excited state.

## 1. Introduction

**Figure 1:**
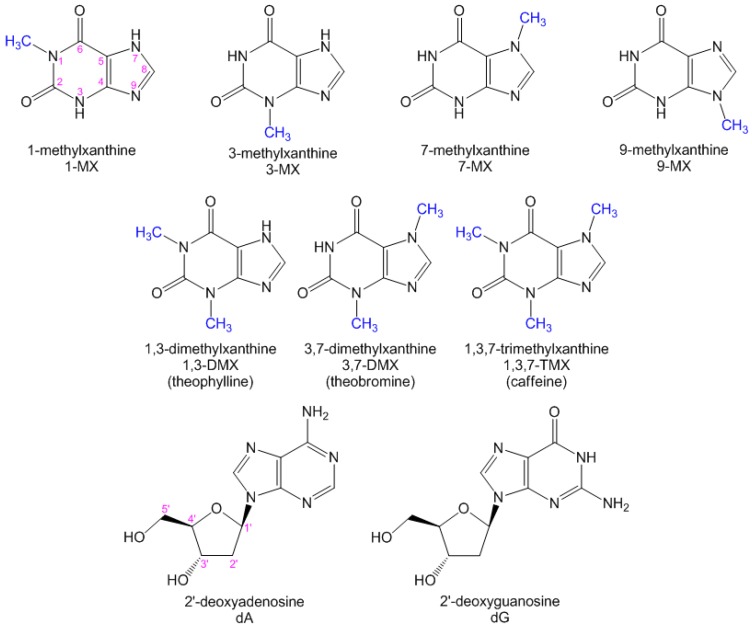
Schematic representation of the studied methylxanthines and of the DNA nucleosides dA and dG. Canonical numbering for the purine ring is noted in the structural formula of 1-methylxanthine, while that of the sugar ring is shown in the structural formula of 2′-deoxyadenosine (dA).

The term “xanthines” denotes a wide class of compounds whose central core (3,7-dihydro-purine-2,6-dione) is closely related to the DNA bases guanine and adenine (Figure 1). Like nucleobases, xanthines are capable of forming hydrogen bonds, allowing their insertion in duplexes [1] and guanine quadruplexes (G-quadruplexes) [2,3,4]. Moreover, it has been shown that their self-association can give rise to four-stranded structures [2,5,6,7,8], which have attracted attention for applications in the field of molecular electronics [9]. Xanthines are ubiquitous in the human body, where they are formed following enzymatic degradation of adenine and guanine. Moreover, methylxanthines are used as therapeutic agents acting, among others, as stimulants of the nervous system [10,11]. The well-known caffeine, present in coffee and tea and various soft beverages, is none other than 1,3,7-trimethylxanthine (Figure 1). It is also worth noticing that 2′-deoxyxanthosine (dX) has been used in the extension of the genetic alphabet by purine pairing with a 2,4-diaminopyrimidine nucleoside, which has a hydrogen bonding pattern complementary to dX [12].

Despite the omnipresence of xanthines in our life, very few studies discuss their intrinsic fluorescence properties. Shukla and Mikla examined the effect of pH on the fluorescence spectra of several xanthines [13]. McKemy et al. reported that the determination of calcium release in cells by measuring the fluorescence signal of the dye indo-1 may be perturbed by the intrinsic fluorescence of caffeine [14]. Karim et al. developed an array for the simultaneous determination of acetylsalicylic acid and caffeine in commercial tablets exploiting their natural fluorescence [15]. Wei et al. studied the fluorescence performance of three methylxanthine derivatives at low and room temperatures and reported fluorescence quantum yields in fluid solutions ranging from 0.2 to 0.6 [16]. Surprisingly, these values are higher by at least three orders of magnitude than those determined for the DNA bases in solution [17]. So far, no xanthine fluorescence lifetime is available in the literature. Therefore, in view of the possible analytical applications, and because of the potential use of xanthines as artificial letters of the genetic code and building blocks of G-quadruplexes, a better understanding of their fluorescence dynamics, and thereby the electronic state relaxation processes, is necessary.

The objective of the present study is to provide a first insight into the dynamical properties of xanthine fluorescence. The main investigation tool is femtosecond fluorescence spectroscopy using fluorescence upconversion, a technique widely proved valuable for the study of DNA bases whose fluorescence is extremely short-lived [17,18]. We examine seven methylated xanthines characterized by different methylation sites and various degrees of methylation (Figure 1), four monomethylated derivatives: 1-methylxanthine (1-MX), 3-methylxanthine (3-MX), 7-methylxanthine (7-MX), and 9-methylxanthine (9-MX); two dimethylated ones: 1,3-dimethylxanthine (1,3-DMX, theophylline) and 3,7-dimethylxanthine (3,7-DMX, theobromine), and one trimethylated compound: 1,3,7-trimethylxanthine (1,3,7-TMX, caffeine).

In the first part, we focus on 3-MX, providing fluorescence spectra and quantum yields in methanol and water as well as fluorescence decays. We compare its behavior with those of the adenine and guanine 2′-deoxynucleosides, dA and dG. Our comparison is made with nucleosides instead of nucleobases because, on the one hand, the solubility of guanine is too low for time-resolved measurements [19] and, on the other hand, the presence of two tautomers renders the study of adenine in solution difficult [20]. Subsequently, we present fluorescence decays and fluorescence anisotropy decays obtained for all the examined compounds in methanol. We chose this solvent for our comparative time-resolved fluorescence measurements because some of the studied xanthines are barely soluble in water, giving rise to aggregates. Finally, we discuss their excited state relaxation in association with the scarce studies tackling this topic, performed by quantum chemistry methods [21,22] and transient absorption spectroscopy [23,24].

## 2. Results

### 2.1. Fluorescence Properties of 3-Methylxanthine

**Figure 2:**
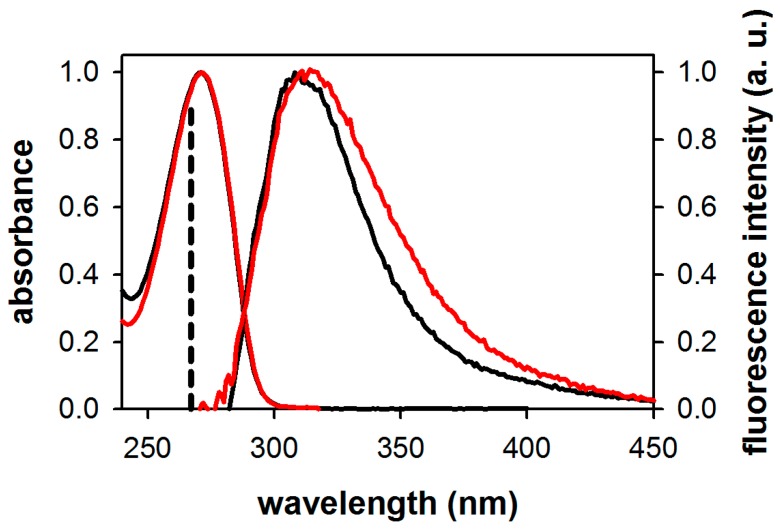
Normalized steady-state absorption and fluorescence spectra of 3-MX in methanol (black) and water (red) on a wavelength scale normalized to their respective maxima. Excitation wavelength: 267 nm (indicated by a vertical dashed line).

Figure 2 shows the steady-state absorption and fluorescence spectra of 3-MX in water and methanol. The absorption spectra in these two solvents are similar, both peaking at 271 nm. The fluorescence spectrum of 3-MX in methanol, which shows a maximum at 311 nm, is slightly blue-shifted from that in aqueous solution, peaking at 315 nm. The spectral halfwidth Δν, determined after conversion of the fluorescence spectra to a wavenumber scale (multiplying by a factor λ^2^), is significantly narrower for 3-MX in methanol (5200 cm^−1^) compared to water (6150 cm^−1^). All these spectral features are gathered in Table 1, together with those of dA and dG in water reported previously [17,25]. The fluorescence quantum yield of 3-MX in water is more than double those of dA and dG determined under the same conditions, but the spectral width is twice as narrow. Note also that 3-MX was found to be about twice as fluorescent in methanol as in water.

**Figure 3:**
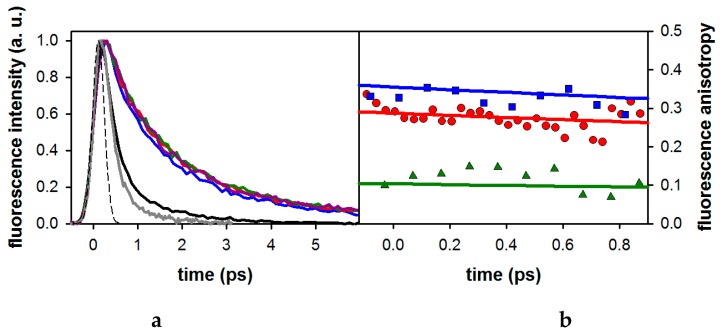
(**a**) Normalized total fluorescence decays recorded at 310 nm (blue), 330 nm (red), 350 nm (violet), and 370 nm (green) for 3-MX in methanol, compared to those recorded for dA (grey; 330 nm) and dG (black; 330 nm) in the same solvent. The instrument response function is displayed as a dashed line. Excitation wavelength: 267 nm. (**b**) Fluorescence anisotropy decays at 330 nm of 3-MX (blue), dA (red), and dG (green) in methanol. Solid lines, derived from fits with mono-exponential functions, are only shown to guide the reader.

Figure 3a displays the total fluorescence decays of 3-MX measured at four wavelengths along with those of dA and dG measured at 330 nm, close to their emission maxima. Also shown in this figure is the 330 fs (fwhm) instrument response function. The fluorescence decays of the xanthine are significantly slower than those of the nucleosides. The dependence of the fluorescence dynamics of 3-MX with the emission wavelength is negligible, except at 310 nm where a slightly faster decay is observed.

The fluorescence anisotropies of 3-MX and the two nucleosides at 330 nm are shown in Figure 3b. The 3-MX anisotropy is somewhat higher than that of dA, while a larger difference is observed with respect to that of dG.

As found for the DNA nucleosides and nucleotides [17,19], the fluorescence decays of 3-MX cannot be fitted by mono-exponential functions. Bi-exponential functions exp(−t/τ_1_) + (1 − α)exp(−t/τ_2_) provided satisfactory fits. The τ_1_ and τ_2_ values are, respectively, in the 0.7–1.11 ps and 2.8–5.5 ps ranges. In Table 2, we compare the average fluorescence lifetimes: <τ> = ατ_1_ + (1 − α)τ_2_) of 3-MX with those of dA and dG derived from the fits of the decays at 330 nm. It appears that the <τ> value of 3-MX in methanol is higher than those of dA (by a factor of 6) and dG (by a factor of 4) in the same solvent. For both nucleotides, when going from methanol to water, the average lifetime decreases. It is also interesting to consider the radiative lifetime τ_rad_, obtained by dividing the fluorescence lifetime by the fluorescence quantum yield [26]. The radiative lifetime is an intrinsic property of the chromophore, directly associated with the oscillator strength, and thereby the electronic structure. This allows a comparison of the different compounds in different environments. Interestingly, the radiative lifetime of 3-MX (3.7 ns) is quite close to that of dG (3.3 ns) but twice as long as that of dA (1.4 ns). All these values correspond, however, to highly allowed transitions.

### 2.2. Substituent Effect on the Fluorescence Dynamics

**Figure 4:**
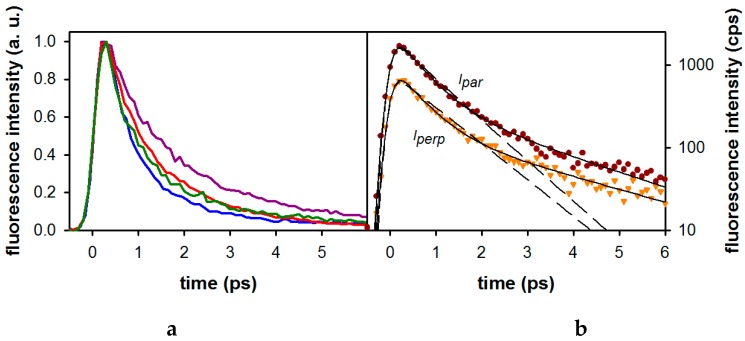
(**a**) Normalized fluorescence decays recorded at 350 nm for monomethylated xanthines in methanol after excitation at 267 nm: 1-MX (blue), 3-MX ((violet), 7-MX (red) and 9-MX (green). (**b**) Parallel and perpendicular fluorescence decays of 1-MX in methanol recorded at 310 nm after excitation at 267 nm. Also shown are the fitted curves using mono-exponential (dashed line) and bi-exponential (solid line) model functions.

**Figure 5:**
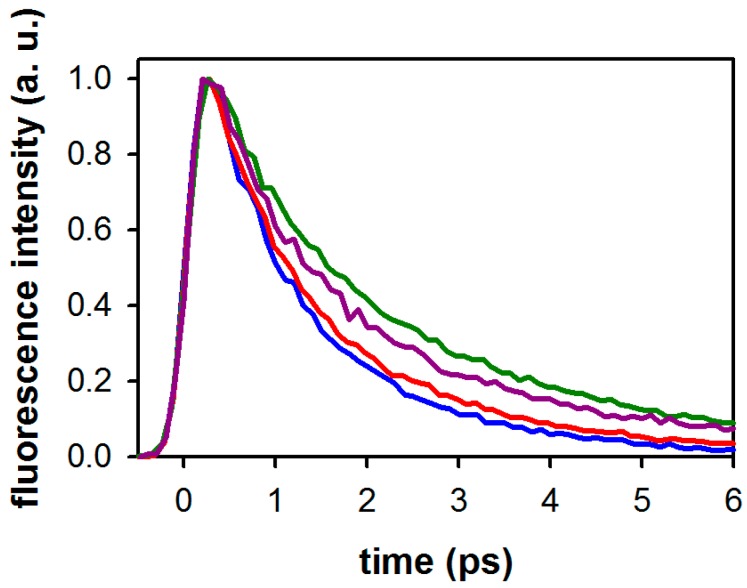
Effect of the degree of methylation on the (normalized) fluorescence decays of xanthines recorded at 350 nm: 3-MX (violet), 1,3-DMX (blue), 3,7-DMX (green), and 1,3,7-TMX (red).

The effect of monomethylation on the fluorescence decays of the xanthine is illustrated in Figure 4a while that of di- and trimethylation is shown in Figure 5. In all cases, good fits required bi-exponential model functions. This is illustrated for 1-MX in Figure 4b. The parameters derived from these fits are given in Table 3.

It appears that, while methylation at position 1 results in the shortest decay (<τ> = 0.91 ps), methylation at position 3 increases the lifetime by a factor of ~2 (<τ> = 1.67 ps). 7-MX and 9-MX exhibit intermediate lifetimes of 1.10 ps and 1.15 ps, respectively. 

Going from monomethylated to dimethylated derivatives, we observe that the <τ> value obtained for 1,3-DMX (1.13 ps) corresponds roughly (within 15%) to the average of the <τ> values (1.29 ps) found for 1-MX (0.91 ps) and 3-MX (1.67 ps). In contrast, the lifetime of 3,7-DMX (2.01 ps) is significantly longer than the average value (1.45 ps) found for the corresponding monomethylated xanthines (1.67 ps for 3-MX and 1.10 ps for 7-MX). Surprisingly, the addition of a third methyl group at position 1 shortens the fluorescence lifetime (<τ> = 1.37 ps for 1,3,7-TMX).

The average fluorescence lifetimes obtained for the di- and trimethylated xanthine derivatives are close to the lifetimes reported by Chen and Kohler [23], also shown in Table 3. The latter were derived from mono-exponential fits of the transient absorption signals obtained for acetonitrile solutions.

**Figure 6:**
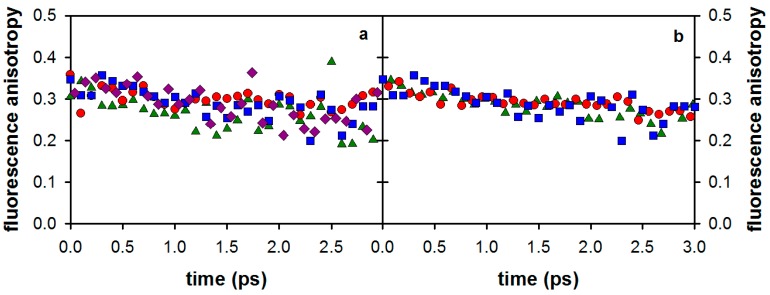
Fluorescence anisotropy decays determined at 350 nm after excitation at 267 nm for (**a**) the monomethylated xanthines: 1-MX (blue), 3-MX (violet), 7-MX (red) and 9-MX (green) and for (**b**) di- and tri-methylated xanthines: 1,3-DMX (blue), 3,7-DMX (red) and 1,3,7-TMX (blue).

The effect of the xanthine monomethylation on the fluorescence anisotropy is shown in Figure 6a while the anisotropy signals of di- and trimethylated xanthines are shown in Figure 6b. As illustrated by the figure, the anisotropies of the four compounds are similar. Comparable anisotropy decays were also observed for the di- and tri-methylated xanthines. The merged nonlinear fitting/deconvolution process of the measured (I_par_) and (I_perp_) signals, as described in the Materials and Methods section, allowed us to extract the time-zero anisotropies r_0_, shown in Table 3. The r_0_ values found for all studied xanthines are around 0.33. Note that, due to the very short fluorescence decays of the studied compounds (<3 ps), it was not possible to extract the rotational diffusion times, which are expected to be about 20–30 ps.

## 3. Discussion

In the first part of our study, we found that the 3-MX absorption spectrum is characterized by a strong band peaking at 271 nm. This corresponds to the first ππ* transition. Quantum chemistry calculations on various methylated xanthines have shown that the next ππ* transition is situated about 1 eV higher [13,22]. Thus, excitation at 267 nm populates only the lowest excited ππ* state of 3-MX. This contrasts with dG, and to a lesser extent dA, for which two near-lying ππ* states are populated at 267 nm. Subsequently, internal conversion between these two ππ* states leads to depolarization faster than our time-resolution (100 fs after deconvolution). Consequently, the time-zero anisotropy determined for the nucleosides (0.15 for dG and 0.24 for dA, see ref. [17]) is lower than the 0.4 value corresponding to parallel absorption and emission dipoles. Yet, the r_0_ value determined for 3-MX, as well as for all the studied xanthine derivatives (0.31–0.34), is still lower than the theoretical limit. Such a discrepancy may be explained by vibrational relaxation in the excited state. A similar effect was noticed for the extensively studied thymine chromophore [27]. 

The fluorescence lifetime of 3-MX in methanol was found to be 1.67 ps, while the radiative lifetime is estimated to about 3.7 ns (1.67 ps/4.5 × 10^−4^, from Table 1 and Table 2). These findings allow us to draw two important conclusions: (i) the emission corresponds to a strongly allowed ππ* electronic transition, as for the DNA mono-nucleosides and mono-nucleotides [17]; (ii) the 3-MX excited state is very efficiently depopulated by non-radiative processes.

Our results are in line with those reported recently from transient absorption measurements [23], showing that all studied methylxanthines undergo ultrafast excited state relaxation. Despite their slightly slower fluorescence decay, their behavior is very similar to that of the DNA/RNA building blocks [18,28]. In the introduction, we evoked the structural similarities between xanthine and the purines adenine/guanine. In Table 2, it is evident that the average florescence decay of 3-MX (a “typical” mono-methylated xanthine) is about five times slower than those of dA and dG. This hints at a possible difference in deactivation mechanism between the xanthine and the two natural DNA building blocks. At the same time, the 1.67 ps time constant of 3-MX corresponds to an “ultrafast” excited state deactivation, which means that the presence of a conical intersection is highly probable. 

Numerous theoretical studies have attributed this ultrafast excited state relaxation to very efficient non-radiative processes involving easily accessible conical intersections (CIs) connecting the first ππ* excited state with the ground state. For a recent review on theoretical studies of the excited states of nucleobases, see [29]. These processes are accompanied by an out-of-plane motion of peripheral substituents, at the 2-position for purines and the 5-position for pyrimidines [18]. A systematic study of the substituent effect had been performed via femtosecond fluorescence spectroscopy for uracil derivatives [30]. Only methylation at the 5-position was found to significantly increase the excited state lifetime (by a factor of 4) of uracil (0.39 ps vs. 0.10 ps) in line with an out-of-plane motion of the C5 carbon [30,31,32,33,34].

As can be seen in Figure 1, the 2-position in xanthines is blocked by a carbonyl group and the 5-position by the imidazole ring. Yet, the average lifetimes reported in Table 3 for monomethylated xanthines show a weak dependence on the methylation site. While the <τ> values of 1-MX, 7-MX, and 9-MX are close to 1 ps (Table 3), that of 3-MX jumps up to 1.7 ps, suggesting that the substitution at the 3-position significantly affects the excited state dynamics. In this regard, it should be recalled that 3-substituted xanthines are potentially tetrad- and G-quadruplex-forming compounds [2].

The effect of the substitution in the 3-position is, however, far from being trivial in di- and trimethylated xanthines. The longest lifetime of 2.0 ps is observed for 3,7-DMX (theobromine), while 1,3-DMX (theophylline) and 1,3,7-TMX (caffeine) exhibit faster decays than that of 3-MX. We stress that, for the monomethylated compounds, methylation at the 1- or the 7-position has very little effect (<20%) on the excited state lifetime. This can be taken as a strong indication that the excited state deactivation of xanthines cannot be reduced to the involvement of one single localized bond. Interestingly, Nachtigallova et al. considered the existence of two different conical intersections in the excited state deactivation of theophylline, theobromine, and caffeine: (i) an out-of-plane deformation of the five-membered imidazole ring, and (ii) an out-of-plane deformation of O6 on the six-membered ring [22]. In addition, direct or indirect Franck-Condon-CI reaction paths were found to be energetically allowed, leading to potentially very complex excited state dynamics. In this context, the peculiar influence of methylation observed above is not surprising. The above calculations predicted reaction paths involving different energy barriers. The smallest barrier was found for 1,3-DMX (0.2 eV), followed by 1,3,7-TMX (0.6 eV) and 3,7-DMX (1.2 eV). This energetic ordering is in excellent agreement with the observed fluorescence lifetimes for these three compounds, 1.13 ps, 1.37 ps, and 2.01 ps, respectively, even though the height of these barriers is not necessarily compatible with picosecond lifetimes. It should also be kept in mind that solvent effects, which were not considered in these calculations, can lower the energy barriers [31].

As already pointed by Chen and Kohler [23], the xanthine structure is closely related to that of uracils. Both molecules contain a heteroatomic 6-membered ring with two carbonyl oxygens in the same positions (positions 2 and 6 for xanthines, positions 2 and 4 for uracils) and one double bond (C4-C5 for xanthine, C5-C6 for uracil). The major difference is the presence of a five-membered imidazole ring attached to the C4-C5 double bond in xanthines. Considering the strong involvement of the C5-C6 double bond in the non-radiative deactivation mechanism of uracils, the little influence of the imidazole ring on the “uracil-like” structure of xanthines is surprising. The excited state dynamics of uracils leading to the conical intersection have often been described as an ethylene-like torsional movement, bringing both the 5- and the 6-substituents out-of-plane in an opposite manner [35]. The “blocking” effect of substitution on the 5-position is today well understood, and explained by the out-of-plane motion of the substituent [30]. Blocking the C5-C6 double bond inhibits efficiently the non-radiative decay of uracils as shown by Lim and coworkers in the case of 5,6-trimethyleneuracil (TMU), for which the excited state lifetime increases to several tens of picoseconds [36]. The non-radiative decay mechanism taking place in the structurally very close molecule cytosine involves a similar C5-C6 activity; by constraining the double bond in 5,6-trimethylenecytosine (TMC), the excited state lifetime increases dramatically from 0.3 ps to 1.2 ns [36]. Such a “blocking” effect is not observed in the case of the investigated methylxanthines. Its absence can be explained by the internal dynamics of the imidazole ring that is largely responsible for the non-radiative decay [22]. 

From the above discussion, it appears that it is difficult to generalize mechanistic schemes between closely related purines and pyrimidines. The decay paths of each heteroatomic system must be treated individually. The fingerprint of the decay path, depending on the topology of the Potential Energy Surface (PES) of the emitting state, is actually reflected in the width of the steady-state fluorescence spectra (Table 1). For both dG [37] and dA [25], quantum chemistry calculations predict a pronounced “flat” and barrierless shape of the PES. This induces a substantial spreading of the wavepacket during its motion towards the conical intersection resulting in a spectral broadening of the fluorescence. For 3-MX, on the other hand, the substantially longer fluorescence lifetime is in line with the presence of a barrier on the PES, confining the excited state population and giving rise to a narrower emission spectrum.

## 4. Materials and Methods

1-Methylxanthine (1-MX) and 9-methylxanthine (9-MX) have been obtained from Molekula GmbH (Garching bei München, Germany), 3-methylxantine (3-MX) from TCI Europe N.V. (Zwijndrecht, Belgium), theophylline (1,3-DMX), theobromine (3,7-DMX), and caffeine (1,3,7-TMX) from Sigma-Aldrich (Budapest, Hungary). 7-Methylxanthine (7-MX) was synthesized by modifying the procedure by Bridson et al. [38]. Thus, guanosine was methylated with methyl iodide and then hydrolyzed with hydrochloric acid. The resulting 7-methylguanine hydrochloride was treated with sodium nitrite in acetic acid, and the product was recrystallized from water (mp. > 360 °C). The ^1^H-NMR spectrum of 7-MX obtained in this way was identical to that described by Müller et al. [39].

Steady-state absorption and fluorescence spectra were recorded with a Perkin-Elmer Lambda 900 spectrophotometer (Bodenseewerk, Perkin-Elmer Gmbh, Überlingen, Germany) and a SPEX Fluorolog-3 spectrofluorometer (Horiba Jobin Yvon Inc., Edison, New Jersay, USA). Methanol for UV spectroscopy was obtained from Fluka (Sigma-Aldrich Chemie GmbH, Steinheim, Germany). Fluorescence quantum yields were determined using TMP (thymidine monophosphate) in water as a reference (Φ_F_ = 1.54 × 10^−4^, λ_exc_ = 255 nm) [17]. TMP was obtained from Sigma-Aldrich (Sigma-Aldrich Chemie GmbH, Steinheim, Germany) and ultrapure water delivered by a Millipore Milli-Q Synthesis Millipore SAS, Molsheim, France) system. 

Time-resolved fluorescence measurements were carried out with the fluorescence up-conversion technique (FU) using the third harmonic (267 nm) of a Ti/sapphire laser system (Coherent MIRA, 76 MHz, 120 fs, 800 nm) for the excitation [27].

The average excitation power was 60 mW. The sample fluorescence was collected with two parabolic mirrors and mixed with the residual 800 nm fundamental beam in a 0.5 mm type I BBO crystal. The up-converted signal was then dispersed in a double-grating monochromator (spectral resolution ca. 5 nm) and detected by a photomultiplier in single photon counting mode. The fluorescence decays were recorded in a 10 ps time window, with parallel and perpendicular excitation-detection polarization configurations by adjusting the excitation beam polarization with a zero-order half-wave plate. 

The total fluorescence kinetics *F*(*t*) shown in Figure 3, Figure 4 and Figure 5 were constructed from the parallel, *I_par_*(*t*), and perpendicular, *I_perp_*(*t*), signals according to the equation $F(t)=I_{par}(t)+2I_{prep}(t)$. Likewise, the fluorescence anisotropy *r*(*t*) is given by the expression: $r(t)=(I_{par}(t)-I_{prep}(t))/F(t)$.

In order to evaluate the characteristic times involved, instead of treating *F*(*t*) and *r*(*t*) separately, we performed a merged nonlinear fitting/deconvolution process using the impulse response model functions $i_{par}(t)=(1+2r(t))f(t)$ and $i_{prep}(t)=(1-r(t))f(t)$, convoluted by the Gaussian instrument response function, *I*(*t*) ∝ *i*(*t*) ⊗ *G*(*t*). The model functions thus obtained were fitted to the experimentally measured *I_par_*(*t*) and *I_perp_*(*t*) signals. The full width at half maximum (fwhm) of the instrument response function was found to be about 350 fs fwhm at 330 nm.

## 5. Conclusions

Our study shows that, in terms of fluorescence, methylated xanthines behave very much as DNA nucleosides and nucleosides. Their room temperature solutions are very weakly emitting, and their fluorescence decays, being extremely short lived, can be determined only using femtosecond techniques. The position and the degree of methyl substitution do not influence dramatically the excited state deactivation rate. However, the fluorescence properties reported in the present work concern xanthines as monomeric chromophores. In environments where their solubility is low, or at high concentrations, self-aggregation may occur, leading to important changes in the nature of the excited states and, consequently, on the fluorescence properties. Indeed, it has been shown that guanine quadruplexes, due to strong electronic interactions among the bases giving rise to collective excited states, exhibit higher fluorescence quantum yields and longer fluorescence lifetimes than isolated guanosines in solutions [40,41,42]. In order to evaluate such effects in the case of xanthines, specific studies focusing on self-associated structures are needed. This is a prerequisite in order to use their fluorescence for analytical purposes. 

## Figures and Tables

**Table 1:** Comparison of the steady-state fluorescence properties of 3-MX in methanol with those of dA and dG in water: fluorescence maximum (λ_fl_), fluorescence quantum yield (φ_f_), and spectral width (Δν).

Compound/Solvent	λ_fl_ (nm)	φ_f_ × 10^4^	Δν (cm^−1^)
3-MX/MeOH	311	4.5	5200
3-MX/H_2_O	315	2.4	6150
dA/H_2_O	307 ^1^	0.9 ^1^	10,150
dG/H_2_O	334 ^1^	1.0 ^1^	11,390

^1^ Onidas et al. [17].

**Table 2:** Comparison of the fluorescence lifetimes of 3-MX in methanol at 350 nm with those of dA and dG in the same solvent at 330 nm: average fluorescence lifetime <τ> and radiative lifetime (τ_rad_).

Compound/Solvent	<τ> (ps) ^1^	τ_rad_ (ns)
3-MX/MeOH	1.67	3.7
dA/H_2_O	0.13 ^2^	1.4
dA/MeOH	0.27 ^3^	nd
dG/H_2_O	0.33 ^4^	3.3
dG/MeOH	0.42 ^3^	nd

^1^ <τ> = ατ_1_ + (1 − α)τ_2_; ^2^ Onidas et al. [17]; ^3^ present work; ^4^ Miannay et al. [19].

**Table 3:** Parameters derived from the fits of the fluorescence decays and anisotropy decays measured at 350 nm after excitation at 267 nm for methylated xanthines in methanol.

Compound	α	τ_1_ (ps)	τ_2_ (ps)	<τ> (ps) ^1^	r_0_	τ_TA_ (ps) ^2^
1-MX	0.82	0.54	2.62	0.91	0.34	
3-MX	0.49	0.66	2.65	1.67	0.33	
7-MX	0.49	0.44	1.72	1.10	0.32	
9-MX	0.68	0.52	2.50	1.15	0.31	
1,3-DMX	0.51	0.60	1.69	1.13	0.33	1.7
3,7-DMX	0.35	0.71	2.70	2.01	0.32	2.0
1,3,7-TMX	0.65	0.85	2.35	1.37	0.33	1.3

^1^ average fluorescence lifetime <τ> = τ_1_ + (1 − α)τ_2_; ^2^ monoexponential decay time observed in acetonitrile by transient absorption [23].
